# Laparoscopic repair of iatrogenic diaphragmatic hernia following pedicled omentoplasty using a modified Sugarbaker technique with round ligament reinforcement

**DOI:** 10.1093/jscr/rjag278

**Published:** 2026-09-25

**Authors:** Yuto Kawate, Akinari Miyazaki, Ryoma Sakamoto, Hiroshi Kusanagi

**Affiliations:** Department of Gastroenterological Surgery, Kameda Medical Center, 929, Higashimachi, Kamogawa, Chiba 2960041, Japan; Department of Gastroenterological Surgery, Kameda Medical Center, 929, Higashimachi, Kamogawa, Chiba 2960041, Japan; Department of Gastroenterological Surgery, Kameda Medical Center, 929, Higashimachi, Kamogawa, Chiba 2960041, Japan; Department of Gastroenterological Surgery, Kameda Medical Center, 929, Higashimachi, Kamogawa, Chiba 2960041, Japan

**Keywords:** iatrogenic diaphragmatic hernia, omentoplasty, mediastinitis, Sugarbaker technique, laparoscopic hernia repair

## Abstract

Iatrogenic diaphragmatic hernia is a rare complication following pedicled omentoplasty for post-cardiac surgery mediastinitis. No standardized repair technique has been established for this condition. We report the case of a 79-year-old man who developed an iatrogenic diaphragmatic hernia with transverse colon herniation into the mediastinum following omentoplasty after aortic arch replacement. We successfully performed a totally laparoscopic repair using a modified Sugarbaker technique with reinforcement of the peri-pedicle area using the round ligament of the liver. At 2-year follow-up, no recurrence was observed. The proposed modifications—dorsal pedicle lateralization and round ligament reinforcement of the peri-pedicle area—may improve mesh stability and reduce recurrence risk in iatrogenic diaphragmatic hernia repair while preserving the omental vascular pedicle.

## Introduction

Pedicled omentoplasty is an effective treatment for mediastinitis following cardiac surgery [1, 2]. However, iatrogenic diaphragmatic hernia through the diaphragmatic opening created for omental transposition is a rare complication without a standardized repair technique [3, 4]. To our knowledge, reinforcement of the peri-pedicle region using the round ligament of the liver during a modified Sugarbaker repair has not been previously described. We report a case of iatrogenic diaphragmatic hernia successfully repaired by a totally laparoscopic approach using a modified Sugarbaker technique with round ligament reinforcement.

## Case report

**Figure 1:**
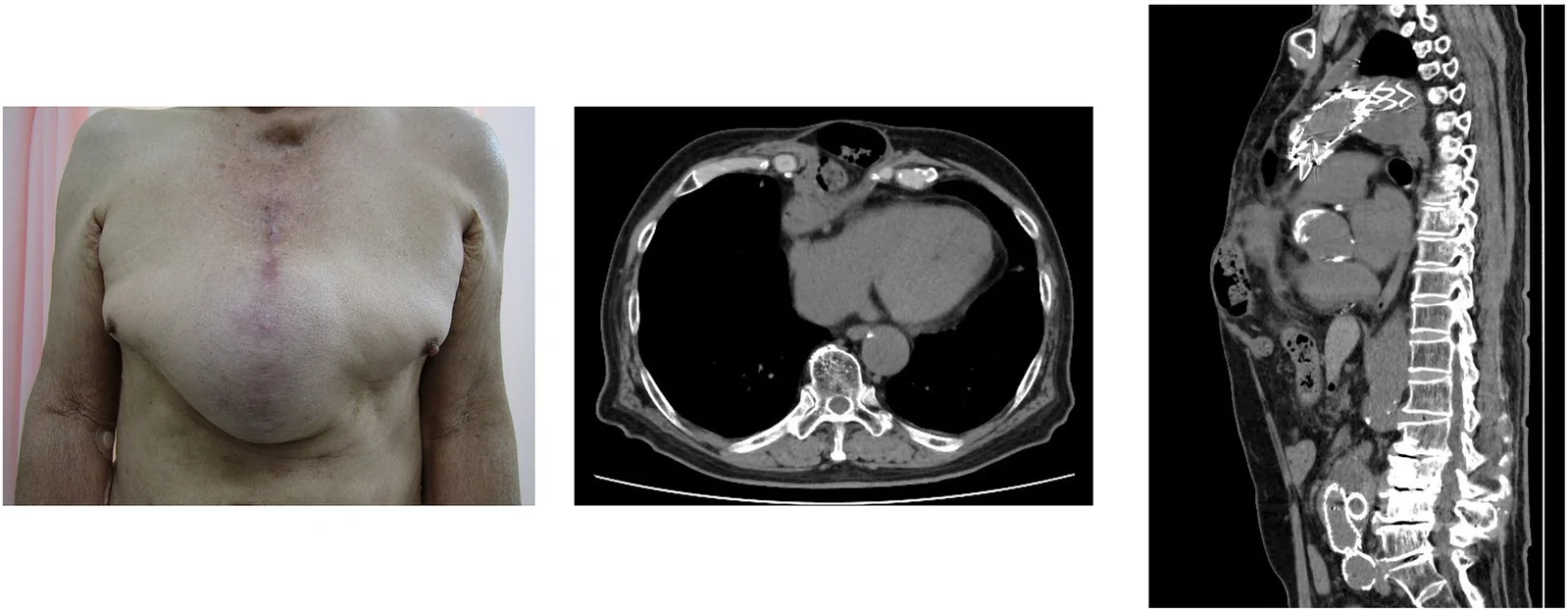
Preoperative findings of iatrogenic diaphragmatic hernia following pedicled omentoplasty. Left: Clinical photograph demonstrating prominent bulging of the anterior chest wall. Center: Axial CT image showing herniation of the transverse colon into the anterior mediastinum. Right: Sagittal CT image confirming the herniation through the diaphragmatic defect.

A 79-year-old man presented with progressive anterior chest wall bulging and exertional dyspnea. Two years prior, he had undergone total arch replacement for aortic arch aneurysm. The postoperative course was complicated by prosthetic graft infection and mediastinitis, treated with surgical debridement, negative pressure wound therapy, and subsequent pedicled omentoplasty using the right gastroepiploic vessels as the vascular pedicle. Computed tomography (CT) revealed herniation of the transverse colon into the anterior mediastinum (Fig. 1). Given progressive symptoms, surgical repair was indicated.

**Figure 2:**
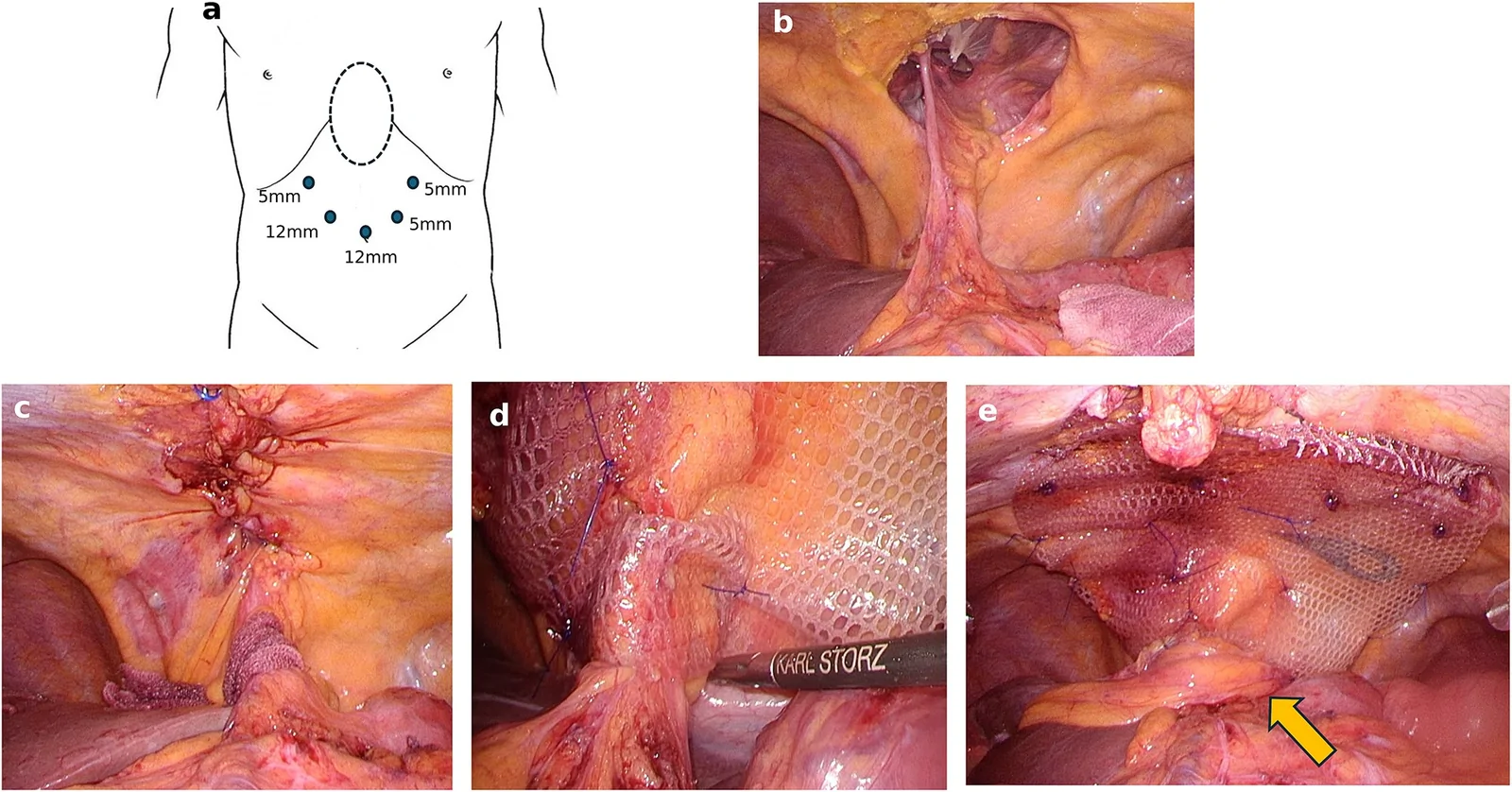
Intraoperative laparoscopic findings and surgical procedure. (a) Schematic illustration of port placement. (b) Intraoperative view demonstrating the hernia defect (5 × 5 cm) with the right gastroepiploic vessels traversing through the hernia orifice. (c) The hernia defect closed with continuous 3-0 V-Loc barbed sutures. (d) Mesh positioning using the Sugarbaker technique; note the mesh elevation around the pedicle entry site caused by tension from the omental pedicle. (e) Final operative view showing the Symbotex Composite Mesh (15 × 10 cm) with the omental vascular pedicle dorsally lateralized and the pedicle entry site reinforced with the round ligament of the liver (arrow).

The patient was placed in the supine position under general anesthesia. A totally laparoscopic approach was employed using a five-port technique (Fig. 2a): a 12-mm camera port at the umbilicus and four working ports (one 12-mm and three 5-mm ports) in the bilateral upper abdomen. Intraoperative findings revealed a 5 × 5 cm hernia defect ventral to the omental vascular pedicle (Fig. 2b). The herniated transverse colon was easily reduced without adhesiolysis. The round and falciform ligaments were divided for mesh placement. The hernia defect was closed with continuous 3-0 V-Loc barbed sutures (Fig. 2c).

A Symbotex Composite Mesh (15 × 10 cm) was placed using the Sugarbaker technique with the omental vascular pedicle dorsally lateralized. The mesh was positioned with at least 5 cm of overlap beyond the defect margins, with the anti-adhesive surface oriented toward the abdominal cavity. Tension from the omental pedicle caused mesh elevation around the pedicle entry site (Fig. 2d); the previously divided round ligament of the liver was used to reinforce and secure the mesh around the pedicle (Fig. 2e). Mesh fixation was achieved with sutures in the central diaphragmatic area where cardiac pulsation was visible and spiral tackers at ~1-cm intervals near the costal margin. The operative time was 180 min with minimal blood loss.

The postoperative course was uneventful except for tacker-related pain that resolved within one week. The patient was discharged on postoperative day 8. CT at 2 years demonstrated no hernia recurrence.

## Discussion

Iatrogenic diaphragmatic hernia following pedicled omentoplasty was first reported by van Garderen *et al*. in 1991 [4], with a reported incidence of ˂5% [5]. Although most cases are asymptomatic, some patients require surgical intervention [6–8]. Reports on repair are limited, and recurrences have been reported [9, 10], highlighting the need for appropriate treatment strategies. The reported surgical techniques and outcomes for this condition are summarized in Table 1; notably, recurrences were observed exclusively in cases repaired using the keyhole technique.

**Table 1:** Summary of reported repairs of iatrogenic diaphragmatic hernia following pedicled omentoplasty.

Author	Technique	Approach	Outcome
Muysoms *et al*. [6]	Sugarbaker-like	Laparoscopic	No recurrence (4 cases)
Hashimoto *et al*. [7]	Keyhole mesh	Open	No recurrence
Matsumoto *et al*. [9]	Keyhole mesh	Open	Recurrence (1 month)
Murata *et al*. [10]	Keyhole mesh	Laparoscopic	Recurrence (2 months)
Present case	Modified Sugarbaker + round ligament	Laparoscopic	No recurrence

The anatomical characteristics of this condition—a subcostal hernia defect with a pedicled omentum traversing the hernia orifice—are analogous to a parastomal hernia. A recent meta-analysis of parastomal hernia repair reported recurrence rates of 24.1% for keyhole, 9% for Sugarbaker, and 3.5% for sandwich techniques [11–13]. In Japan, three mesh-based repairs have been reported using the keyhole technique [7, 9, 10]; however, recurrences occurred in two cases [9, 10], underscoring the limitations of this approach.

Muysoms *et al*. reported favorable outcomes in four cases treated with laparoscopic repair using a technique similar to the Sugarbaker method [6]. However, whereas Muysoms *et al*. lateralized the pedicle to the left, requiring sufficient pedicle length, our technique positions the pedicle dorsally and secures it against the posterior chest wall with the mesh (dorsal lateralization), enabling successful repair even with limited pedicle length.

Furthermore, the peri-pedicle area tends to become unstable due to tension from the omental pedicle. To reinforce this vulnerable area, we utilized the round ligament of the liver, ensuring secure coverage of the pedicle entry site. Although the Sugarbaker principle has been previously applied to this condition [6], the present report introduces two technical modifications: dorsal lateralization of the pedicle and round ligament reinforcement of the peri-pedicle area. These modifications may broaden the applicability of this repair strategy.

Regarding mesh fixation, Köckerling *et al*. reported 25 cases of serious cardiac injury due to tacker use on the diaphragm, with 12 fatalities [14]. Therefore, suture fixation is recommended in areas where cardiac pulsation is observed.

We also performed primary closure of the hernia defect before mesh placement to reduce the hernia orifice and minimize dead space. This is consistent with the IntraPeritoneal Onlay Mesh-plus principle, in which fascial closure combined with mesh overlay has been shown to reduce recurrence compared with bridging repair alone [15].

This study has several limitations. As a single case report, no comparative analysis with alternative techniques was possible, and generalizability is limited. Although 2-year follow-up demonstrated no recurrence, longer-term durability remains uncertain, particularly given that late recurrences beyond 5 years have been reported in parastomal hernia repair. Extrapolation of parastomal hernia data warrants caution given the distinct biomechanical environment of the diaphragm. Additionally, the biomechanical benefit of round ligament reinforcement has not been validated experimentally, and its long-term tensile strength remains unknown. Further accumulation of cases is warranted to validate this approach.

In conclusion, we achieved favorable outcomes using a modified Sugarbaker technique with round ligament reinforcement for iatrogenic diaphragmatic hernia following omentoplasty. This approach may provide a safe option for institutions experienced in laparoscopic hernia repair.
